# Treatment patterns for ductal carcinoma *in situ* from 2000–2010 across six integrated health plans

**DOI:** 10.1186/s40064-014-0776-7

**Published:** 2015-01-17

**Authors:** Heather Spencer Feigelson, Nikki M Carroll, Sheila Weinmann, Reina Haque, Chu-Ling Yu, Melissa G Butler, Beth Waitzfelder, Michelle G Wrenn, Angela Capra, Elizabeth A McGlynn, Laurel A Habel

**Affiliations:** Institute for Health Research, Kaiser Permanente, Denver, CO USA; Center for Health Research, Kaiser Permanente Northwest, Portland, OR USA; Kaiser Permanente Southern California, Pasadena, CA USA; Mid-Atlantic Permanente Research Institute, Kaiser Permanente, Rockville, MD USA; Kaiser Permanente Georgia, Atlanta, GA USA; Center for Health Research, Kaiser Permanente Hawaii, Honolulu, HI USA; Kaiser Permanente Division of Research, Oakland, CA USA; Kaiser Permanente Center for Effectiveness and Safety Research, Pasadena, CA USA

**Keywords:** Breast cancer, Ductal carcinoma in situ, Treatment, Mastectomy, Race

## Abstract

Considerable debate exists about the optimal treatment of ductal carcinoma *in situ* (DCIS). Using electronic data sources, we examined first course treatment patterns among women aged 18 years and older diagnosed with DCIS between 2000–2010 from six Kaiser Permanente (KP) regions. We calculated the proportion of patients receiving breast conserving surgery (BCS), BCS plus radiation therapy, unilateral mastectomy, bilateral mastectomy, and hormone therapy. Multinomial logistic regression was used to assess the association between patient characteristics and treatment. We included 9,437 women: 1,086 (11.5%) African-American; 1,455 (15.4%) Asian; 918 (9.7%) Hispanic; and 5,978 (63.3%) non-Hispanic white. Most cases (42.2%) received BCS plus radiation as their initial treatment. Nearly equal numbers of women received BCS without radiation (28.5%) or unilateral mastectomy (24.6%). Use of bilateral mastectomy was uncommon (4.7%), and most women (72.2%) did not receive hormone therapy has part of their first course treatment. We observed statistically significant differences in treatment patterns for DCIS by KP region and patient age. Predictably, nuclear grade and the presence of comorbidities were associated with first course treatment for DCIS. We observed statistically significant increases in BCS plus radiation therapy and bilateral mastectomy over time. Although still uncommon, the frequency of bilateral mastectomy increased from 2.7% in 2000 to 7.0% in 2010. We also observed differences in treatment by race/ethnicity. Our findings help illustrate the complex nature of DCIS treatment in the United States, and highlight the need for evidence based guidelines for DCIS care.

## Background

Ductal carcinoma *in situ* (DCIS) describes breast lesions characterized by proliferation of abnormal epithelial cells with an intact basement membrane and no evidence of stromal invasion. While the age-adjusted incidence of invasive breast cancer remained relatively stable in the 1980s and 1990s, the incidence of a DCIS diagnosis rose rapidly, largely as a result of increased mammography screening (American Cancer Society 2014; Virnig et al. 2010). Since 1999, the rates have stabilized in women age 50 and older but have continued to increase in younger women. It is estimated that about 22% of all new breast cancers in 2013 were DCIS (Siegel et al. 2013).

DCIS is non-fatal; however, it is considered to be a precursor to invasive cancer and it is unclear which women will develop invasive cancer (Jackson et al. 2008). Considerable debate exists about the optimal treatment for DCIS, and many have expressed concern that DCIS is over diagnosed and over treated. The 2009 National Institutes of Health State of the Science Conference even recommended that “strong consideration should be given to elimination of the use of the anxiety-producing term ‘carcinoma’ from the description of DCIS” (Allegra et al. 2010). This has led some to suggest that it may be more appropriate to adopt a prophylactic approach to managing patients who present with DCIS, similar to the approach for managing patients with lobular carcinoma *in situ* of the breast (Punglia et al. 2013). Prophylactic treatment of DCIS after excision would aim to decrease the risk of development of invasive cancer, rather than to eradicate residual disease or reduce recurrence.

Given the frequency of a DCIS diagnosis, and the debate about how to best manage these cases, it is not surprising that many treatment options are available for women diagnosed with DCIS. Using the Kaiser Permanente (KP) electronic databases and tumor registries, we examined first course treatment patterns among women diagnosed with DCIS from January 1, 2000 through December 31, 2010 across six KP regions: Colorado, Georgia, Hawaii, Northern California, Northwest, and Southern California. The purpose of this analysis was to describe DCIS treatment across these community based health plans and over time and to identify factors that may influence treatment decisions.

## Methods

### Data sources

The primary data source for this study was the KP Center for Effectiveness and Safety Research Virtual Data Warehouse (VDW). As described previously, the VDW includes standardized variables derived from administrative databases at each KP site (Ross et al. 2014; Ritzwoller et al. 2013; Hornbrook et al. 2005). Within the VDW, the Virtual Tumor Registry (VTR) contains data consistent with the North American Association of Central Cancer Registries standards (North American Association of Central Cancer Registries 2014). VTR data are derived from manual reviews of cancer patients’ medical charts by trained abstractors. VTR variables include date of diagnosis, first-course definitive treatment (surgery, radiotherapy, chemotherapy, and hormone therapy), tumor characteristics, and patient demographic characteristics. VDW diagnosis and procedure files include coded diagnoses and procedures associated with inpatient and outpatient encounters or events that were extracted from electronic medical records and other claims databases. Codes are based on International Classification of Diseases, 9th Revision, Clinical Modification (ICD-9-CM), Healthcare Common Procedure Coding System (HCPCS), and the Fourth Edition of the Common Procedure Terminology codes (CPT-4). This study was approved by the Institutional Review Boards of the six participating health plans.

### Study population

All women in the VTR aged ≥ 18 years diagnosed with DCIS from 01/01/2000 through 12/31/2010 were identified. The study sample was limited to women for whom this was their first cancer diagnosis (since history of cancer may affect treatment decisions for the current DCIS diagnosis), did not have a simultaneous diagnosis of an invasive breast cancer (defined as an invasive cancer diagnosed with 30 days of the DCIS diagnosis), and were enrolled in the health plan for at least 12 months before and after the DCIS diagnosis.

Patient characteristics of interest including age at DCIS diagnosis, year of DCIS diagnosis, nuclear grade of tumor, and race/ethnicity (White, African American, Hispanic, Asian, Other/Unknown) were collected from the VTR for each eligible woman. As an indicator of general health, we used the Quan adaptation of the Charlson Comorbidity Index modified to exclude cancer diagnoses (Quan et al. 2005) derived from diagnosis codes captured from all hospital and ambulatory encounters that occurred 12 months prior to DCIS diagnosis. Surrogate patient-level measure of socioeconomic status was obtained from VDW 2000 Census files by mapping median education level of census track to patient address.

### Statistical analysis

We calculated the proportion of patients receiving any of the following treatments: breast conserving surgery (BCS), unilateral mastectomy, bilateral mastectomy, BCS plus radiation therapy, and hormone therapy as defined through the VTR first course therapy variables (typically the first six months post diagnosis). Women with no record of surgery, hormone therapy or radiotherapy were classified as having received no treatment.

Differences in the distribution of baseline characteristics between women receiving the different treatments of interest were evaluated using the Wilcoxon rank-sum test for interval-level data and the chi-square test for nominal/ordinal level data. Multinomial logistic regression was used to assess the association between treatment course and the following list of predictors: KP region, year of diagnosis (as a continuous variable), nuclear grade (low/intermediate, high, or unknown), patient age at diagnosis (<50 years, 50 – 59 years, 60 – 69 years, and 70 or more years of age), 2000 census level median education, comorbidity status, and race/ethnicity. Customary residual and influential statistics were examined to assess model fit and overly influential covariate patterns. All analyses were performed using SAS 9.2 (SAS Software Inc., Cary, NC).

## Results

A total of 13,827 women 18 years of age or older were diagnosed with DCIS (with no simultaneous diagnosis of invasive cancer) across the six KP regions from 2000–2010. We excluded 262 women who had a prior diagnosis of DCIS, 1838 women who did not meet our inclusion criteria of KP membership 12 months before and after diagnosis, and 1878 women who had a prior diagnosis of other cancer. We excluded 70 women because their reported treatment was not consistent with DCIS (e.g., chemotherapy); these women could have progressed to invasive disease or developed another cancer during the year following diagnosis of DCIS. It is also possible that these data were in error, or that their treatment was incorrectly recorded. We also excluded 32 women whose first course of therapy data were missing and 138 women who did not receive any treatment. Finally, we excluded 172 women for whom we could not identify their race/ethnicity. Our final dataset included 9,437 women.

Table 1 shows the demographic characteristic of the study population. Our data set includes 1086 (11.5%) African-American women, 1455 (15.4%) Asian women (including Hawaiian and Pacific Islanders), 918 (9.7%) Hispanic women, and 5978 (63.3%) non-Hispanic white women. This distribution reflects the underlying population for the KP regions combined. Most cases were diagnosed between 50–69 years of age (59.0%). The Charlson co-morbidity index, an indicator of prevalent co-morbid conditions, suggests a relatively healthy population at the time of diagnosis: approximately 91% of cases had 0 or 1 concurrent chronic conditions in the year prior to diagnosis. Fifty percent of the tumors were classified as low or intermediate nuclear grade, 40% were high nuclear grade, and for 11% of cases these data were unavailable. Most cases (42.2%) received BCS plus radiation as their initial treatment. Nearly equal numbers of women received BCS without radiation (28.5%) or unilateral mastectomy (24.6%). Bilateral mastectomy was relatively uncommon as first course treatment (4.7%), and most women (72.2%) did not receive hormone therapy has part of their first course treatment.

**Table 1:** **Characteristic of DCIS patient population from six Kaiser Permanente regions, 2000–2010 (N = 9437)**

**Characteristic**	**Number**	**Percent**
Race/Ethnicity		
White	5978	63.3
African American	1086	11.5
Asian	1455	15.4
Hispanic	918	9.7
Year of Diagnosis		
2000-2002	2043	21.6
2003-2004	1650	17.5
2005-2006	1837	19.5
2007-2008	2001	21.2
2009-2010	1906	20.2
Age at Diagnosis		
<50 years	1978	21.0
50 – 59 years	2917	30.9
60 – 69 years	2651	28.1
> = 70 years	1891	20.0
Comorbidity Index		
0	6898	73.1
1	1654	17.5
2+	885	9.4
Nuclear Grade		
Low/Intermediate	4702	49.8
High	3727	39.5
Unknown	1008	10.7
First course surgical therapy		
Breast conserving surgery (BCS)	2689	28.5
BCS + radiation	3978	42.2
Bilateral mastectomy	448	4.7
Unilateral mastectomy	2322	24.6
Hormone Therapy*		
Yes	2625	27.8
No	6812	72.2

*Receipt of hormone therapy in addition to surgical treatment.

**Figure 1:**
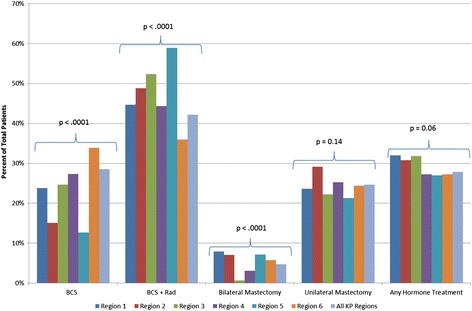
**First course therapy by Kaiser Permanente region.** Each region is represented by a different colored bar, as indicated in the legend; all regions combined shown on the right most bar for each type of treatment. P-values shown for chi-squared test for differences across regions. The “any hormone treatment” group is not mutually exclusive, as patients may also be represented in one of the surgical treatment groups.

We observed regional variation for receipt of BCS, BCS plus radiation, and bilateral mastectomy as first course therapy for DCIS (Figure 1). The fraction of BCS without radiation therapy ranged between 13-34% (p < 0.001), and BCS plus radiation therapy ranged between 36-59% (p < 0.001). Bilateral mastectomy was rare at all sites, and ranged between <1 - 8% (p < 0.001). We did not observe statistically significant variation by region for unilateral mastectomy (p = 0.14) or hormone therapy (p = 0.06) across regions.

**Figure 2:**
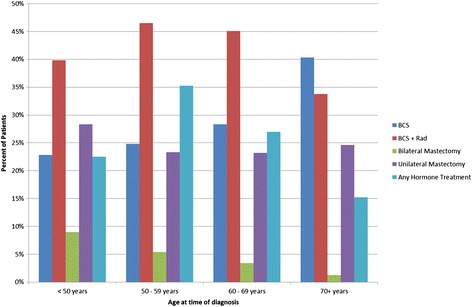
**First course therapy by age group.** Each treatment type is represented by a different colored bar, as indicated in the legend. The “any hormone treatment” group is not mutually exclusive, as patients may also be represented in one of the surgical treatment groups.

We also observed variation in treatment by age (Figure 2). For example, BCS with radiation therapy was the most common treatment among women under age 70 (range 40% - 47%) and BCS without radiation therapy was the most common treatment (40%) among women 70 years and older. Frequency of bilateral mastectomy decreased steadily with increasing age; 9% of women under 50 years received bilateral mastectomy, compared to only 1% of women aged 70 years or older. Frequency of unilateral mastectomy was relatively constant across age groups, and receipt of adjuvant hormone therapy was highest (35%) among women aged 50–59 years.

Table 2 shows the results of multinomial logistic regression for predictors of surgical and radiation treatments, using BCS only as the referent group. The variation in treatment by site observed in Figure 1 remained statistically significant in the multinomial model for all surgical options. Age, Hispanic ethnicity, nuclear grade, comorbidity index and year of diagnosis were also statistically significant predictors of BCS plus radiation therapy compared to BCS alone. Women aged 70 and older were half as likely to have BCS plus radiation therapy (OR: 0.54, 95% CI: 0.45-0.63, p < .0001), while women of Hispanic ethnicity were 30% more likely to have BCS plus radiation therapy (OR:1.30, 95% CI: 1.08-1.56, p = 0.005). Women with high nuclear grade tumors were twice as likely to have adjuvant radiation therapy (OR: 2.01, 95% CI: 1.80-2.25, p < 0.0001), and women with two or more comorbidities were less likely to have BCS plus radiation therapy compared to BCS alone (OR: 0.75, 95% CI: 0.62-0.89, p = 0.002). Likelihood of BCS plus radiation therapy also increased over time (OR: 1.06 per year, 95% CI: 1.04-1.08, p < 0.0001) compared to BCS alone.

**Table 2:** **Odds ratios and 95% confidence intervals from multinomial logistic regression models comparing first course therapy groups with breast conserving surgery (BCS) as referent group**

**Characteristic**	**BCS (N = 2689)**	**BCS + radiation (N = 3978)**	**Unilateral mastectomy (N = 2322)**	**Bilateral mastectomy (N = 448)**
KP Region				
1	1.0	1.20 (0.97-1.49)	1.16 (0.91-1.48)	2.76 (1.88-4.04)
2	1.0	2.10 (1.47-2.99)	2.19 (1.49-3.22)	3.69 (2.07-6.59)
3	1.0	1.31 (0.96-1.78)	0.75 (0.52-1.08)	0.32 (0.08-1.33)
4		[Referent]	[Referent]	[Referent]
5	1.0	3.00 (2.24-4.02)	1.93 (1.39-2.70)	4.59 (2.87-7.34)
6	1.0	0.62 (0.56-0.70)	0.75 (0.66-0.85)	1.39 (1.09-1.78)
Age at diagnosis				
<50 years		[Referent]	[Referent]	[Referent]
50-59 years	1.0	1.12 (0.96-1.30)	0.79 (0.67-0.93)	0.54 (0.42-0.70)
60-69 years	1.0	0.98 (0.84-1.14)	0.69 (0.59-0.82)	0.29 (0.22-0.39)
70+ years	1.0	0.54 (0.45-0.63)	0.55 (0.46-0.65)	0.08 (0.05-0.13)
Race/Ethnicity				
White		[Referent]	[Referent]	[Referent]
African American	1.0	0.88 (0.74-1.04)	0.91 (0.75-1.10)	0.56 (0.39-0.80)
Asian	1.0	1.11 (0.95-1.30)	1.62 (1.37-1.91)	0.52 (0.36-0.76)
Hispanic	1.0	1.30 (1.08-1.56)	1.16 (0.94-1.42)	0.69 (0.48-1.00)
Census Tract % College Educated (Quintiles)				
1: 32.3% - 78.8%	1.0	1.11 (0.94-1.30)	0.99 (0.83-1.19)	1.06 (0.78-1.46)
2: 78.9% - 86.3%	1.0	1.06 (0.90-1.24)	0.77 (0.64-0.92)	0.78 (0.56-1.08)
3: 86.5% - 92.2%	1.0	1.05 (0.89-1.24)	0.88 (0.73-1.05)	0.82 (0.59-1.14)
4: 92.3% - 95.7%	1.0	1.00 (0.84-1.18)	0.76 (0.63-0.92)	0.75 (0.53-1.05)
5 (highest): > 95.7%		[Referent]	[Referent]	[Referent]
Year of Diagnosis	1.0	1.06 (1.04-1.08)	1.00 (0.99-1.02)	1.16 (1.12-1.20)
Comorbidity Index				
0		[Referent]	[Referent]	[Referent]
1	1.0	0.84 (0.73-0.96)	1.01 (0.87-1.18)	0.88 (0.66-1.17)
2+	1.0	0.75 (0.62-0.89)	0.95 (0.78-1.16)	0.81 (0.53-1.23)
Nuclear Grade				
Low/Intermediate		[Referent]	[Referent]	[Referent]
High	1.0	2.01 (1.80-2.25)	2.70 (2.39-3.06)	2.52 (2.03-3.14)
Unknown	1.0	0.93 (0.79-1.10)	1.16 (0.96-1.40)	1.07 (0.73-1.57)

Statistically significant predictors of unilateral mastectomy compared to BCS alone included age, race/ethnicity, education, and tumor grade (Table 2). Compared to the pattern observed for BCS alone, women 50 years of age or older were less likely than women less than 50 years of age to receive unilateral mastectomy, and compared to whites, Asian women were more likely to have unilateral mastectomy (OR: 1.62, 95% CI: 1.37-1.91, p < 0.0001). Women with high nuclear grade tumors compared to low or intermediate nuclear grade were more than twice as likely to receive unilateral mastectomy (OR: 2.70, 95% CI: 2.39-3.06, p < 0.0001). Similarly, women with bilateral mastectomy had many of the same statistically significant predictors as those who underwent unilateral mastectomy including age, race/ethnicity, and tumor grade. However, women of Asian ethnicity were less likely to have bilateral mastectomy compared to white women (OR: 0.52, 95% CI: 0.36-0.76, p = 0.0007). African-American women were also less likely to have bilateral mastectomy (OR: 0.56, 95% CI: 0.39-0.80, p = 0.0014). Likelihood of bilateral mastectomy increased over time compared to BCS (OR: 1.16 per year, 95% CI: 1.12-1.20, p < 0.0001). Although still uncommon, the fraction who underwent bilateral mastectomy increased from 2.7% in 2000 to 7.0% in 2010 (data not shown).

Table 3 compares women with and without adjuvant hormone therapy (initiated in the first six months after diagnosis), regardless of the type of surgical treatment they received. Unlike the surgical and radiotherapy treatments, there was little variation by KP region in the use of hormone therapy. Hormone therapy use declined by age; women aged 60 years and over were less likely than younger women to receive hormone therapy. For women aged 70 years or over, the OR = 0.65 (95% CI: 0.56-0.76, p < .0001) for receipt of hormone therapy compared to women < 50 years of age. African-American women were less likely than white women to receive hormone therapy (OR = 0.82, 95% CI: 0.70-0.96, p = 0.014), while Hispanic (OR = 1.20, 95%CI: 1.02-1.40, p = 0.03) and Asian (OR = 1.18, 95% CI: 1.03-1.34, p = 0.02) women were more likely than white women to receive hormone therapy. Women with two or more comorbidities were less likely to receive hormone therapy (OR = 0.76, 95% CI: 0.64-0.91, p = 0.003), as were women with high nuclear grade or undifferentiated tumors compared to those with low or intermediate nuclear grade tumors (OR = 0.83, 95% CI: 0.76-0.92, p = 0.0002).

**Table 3:** **Odds ratios and 95% confidence intervals from logistic regression model comparing women who received hormone therapy (in addition to surgical therapy) to women who received no hormone therapy**

	**No hormone therapy (N = 6812)**	**Hormone therapy (N = 2625)**
KP region		
1	1.0	1.29 (1.08-1.55)
2	1.0	1.21 (0.93-1.58)
3	1.0	1.14 (0.87-1.49)
4		Referent
5	1.0	0.96 (0.78-1.19)
6	1.0	0.97 (0.87-1.07)
Age at diagnosis		
< 50 years		Referent
50-59 years	1.0	1.11 (0.98-1.25)
60-69 years	1.0	0.88 (0.77-1.00)
70+ years	1.0	0.65 (0.56-0.76)
Race/Ethnicity		
White		Referent
African American	1.0	0.82 (0.70-0.96)
Asian	1.0	1.18 (1.03-1.34)
Hispanic	1.0	1.20 (1.02-1.40)
Census Tract % College Educated (Quintiles)		
1: 32.3% - 78.8%	1.0	1.03 (0.89-1.18)
2: 78.9% - 86.3%	1.0	1.02 (0.88-1.18)
3: 86.5% - 92.2%	1.0	0.96 (0.83-1.11)
4: 92.3% - 95.7%	1.0	0.81 (0.69-0.94)
5 (highest): > 95.7%		Referent
Year of Diagnosis	1.0	0.99 (0.97-1.00)
Comorbidity Index		
0		Referent
1	1.0	1.22 (1.09-1.38)
2+	1.0	0.76 (0.64-0.91)
Nuclear Grade		
Low/Intermediate		Referent
High	1.0	0.83 (0.76-0.92)
Unknown	1.0	0.89 (0.77-1.05)

## Discussion

We observed statistically significant differences in treatment patterns for DCIS by both KP region and patient age. Predictably, we observed that nuclear grade and the presence of comorbidities were associated with first course treatment for DCIS. Because we have large numbers of patients from several racial/ethnic groups, we were able to demonstrate differences in treatment by race/ethnicity. We also observed increases in BCS plus radiation therapy and bilateral mastectomy over time.

Our population’s patterns of treatment with surgery and radiation therapy are similar to those of Liu et al. (2014), who used treatment data from the SEER 18 Registries database, and to Haque et al. (2010), who examined 3000 DCIS cases diagnosed between 1990–2001 in three integrated health plans, two of which were KP locations and included in the current study. Nearly 30% of our patient population were treated with adjuvant hormone agents, which is higher than reported in previous studies (Jackson et al. 2008; Haque et al. 2010; Habel et al. 2009). The increased use of hormone therapy in our study population, who were diagnosed 2001–2010, may be attributed to the clinical trials data that emerged post 2000 that demonstrated tamoxifen’s efficacy in reducing subsequent breast cancer among DCIS patients (Fisher et al. 2001).

Haque et al. (2010), observed no statistically significant differences in adjuvant treatments (radiation therapy and/or hormone therapy) by race/ethnicity; however, we observed several differences in first course treatment by race/ethnicity. Asian women were less likely to have bilateral mastectomy (OR = 0.52, 95% CI: 0.36-0.76, p = 0.0007), and more likely to have unilateral mastectomy (OR = 1.62, 95% CI: 1.37-1.91, p < 0.0001) than white women. African-American women were also less likely to have bilateral mastectomy (OR = 0.56, 95% CI: 0.39-0.80, p = 0.0014) compared to white women. For hormone therapy, Asian and Hispanic women were both more likely to receive hormone therapy, while African American women were less likely to receive hormone therapy compared to white women. It is possible that the proportion of African-American women receiving hormone therapy was lower than white women because African-American women are more likely than white women to have tumors that are estrogen receptor negative (Liu et al. 2014; Howlader et al. 2014). However, receptor status was not reliably captured for our DCIS cases until recently, and thus we could not include receptor status in our analysis. Women with high nuclear grade tumors were also less likely to receive hormone therapy compared to those with low or intermediate grade tumors (OR = 0.83, 95% CI: 0.76-0.92). It is possible that high nuclear grade tumors are also less likely to be estrogen receptor positive, which could explain this association, but we cannot examine this in our data.

The strengths of our study include its geographic variation, large size and racial/ethnic diversity. Our study included over 1,000 African American women, over 900 Hispanic women, and nearly 1,400 Asian women. We have previously demonstrated that the VDW is highly accurate for the report of chemotherapy (Delate et al. 2012); however, the accuracy of radiation therapy is less clear. Other limitations of our study include lack of information on family history, genetic testing, and patient or physician concerns that certainly influence treatment decisions (Arvold et al. 2011; Courdi et al. 2010; Field et al. 2011; Fisher et al. 2012). Further, because we relied on electronic data that were available at all the participating sites over a 10-year period, we are missing information on tumor characteristics not available at all sites, such as size and histology, which also predict treatment choice (Virnig et al. 2010; Silverstein & Lagios 2010; Schmale et al. 2012). Nonetheless, our results are valuable in describing current treatment patterns in the community setting that likely can be generalized beyond Kaiser Permanente.

Our findings help illustrate the complex nature of DCIS treatment in the United States. Our observation of treatment differences, even among regions of the same integrated health care system, is not surprising, given that no current consensus exists on how best to treat DCIS (Allegra et al. 2010; Punglia et al. 2013; Greenberg et al. 2014), and treatment decisions are influenced by patient or physician preference (Morrow et al. 2009), as well as other factors such as distance to radiation therapy facilities. Randomized trials and observational studies have shown that adjuvant radiation therapy reduces the risk of a second ipsilateral event compared to BCS alone (Greenberg et al. 2014). However, there is no evidence that BCS alone results in poorer survival compared to BCS plus radiation therapy, even in the presence of adverse prognostic factors (Virnig et al. 2010). Until we have better clinical or molecular markers to indicate which DCIS patients are most likely to have a subsequent cancer, defining the “best” treatment for an individual woman will remain a challenge.
